# Perceived Weight Discrimination and Obesity

**DOI:** 10.1371/journal.pone.0070048

**Published:** 2013-07-24

**Authors:** Angelina R. Sutin, Antonio Terracciano

**Affiliations:** Florida State University College of Medicine, Tallahassee, Florida, United States of America; Pennington Biomedical Research Center, United States of America

## Abstract

Weight discrimination is prevalent in American society. Although associated consistently with psychological and economic outcomes, less is known about whether weight discrimination is associated with longitudinal changes in obesity. The objectives of this research are (1) to test whether weight discrimination is associated with risk of becoming obese (Body Mass Index≥30; BMI) by follow-up among those not obese at baseline, and (2) to test whether weight discrimination is associated with risk of remaining obese at follow-up among those already obese at baseline. Participants were drawn from the Health and Retirement Study, a nationally representative longitudinal survey of community-dwelling US residents. A total of 6,157 participants (58.6% female) completed the discrimination measure and had weight and height available from the 2006 and 2010 assessments. Participants who experienced weight discrimination were approximately 2.5 times more likely to become obese by follow-up (OR = 2.54, 95% CI = 1.58–4.08) and participants who were obese at baseline were three times more likely to remain obese at follow up (OR = 3.20, 95% CI = 2.06–4.97) than those who had not experienced such discrimination. These effects held when controlling for demographic factors (age, sex, ethnicity, education) and when baseline BMI was included as a covariate. These effects were also specific to weight discrimination; other forms of discrimination (e.g., sex, race) were unrelated to risk of obesity at follow-up. The present research demonstrates that, in addition to poorer mental health outcomes, weight discrimination has implications for obesity. Rather than motivating individuals to lose weight, weight discrimination increases risk for obesity.

## Introduction

There is a pervasive stereotype about obesity in American society: People who are obese are often perceived as lazy, unsuccessful, and weak-willed [1]. These beliefs about individuals with obesity are often translated into negative attitudes [2], discrimination [3], and verbal and physical assaults [4]. Such bias can have severe psychological consequences, including increased vulnerability to depression [5], [6] and lower self-esteem [5], [7], self-acceptance [3], and life satisfaction [8]. A broad range of research now demonstrates that the effects of weight bias are not limited to psychological functioning but extend to nearly every aspect of an individual’s life, from employment [9], [10] and salary disparities [11], [12] to personal relationships [13] to healthcare delivery [14], [15]. In addition, as with other forms of discrimination [16], [17], weight discrimination may have consequences for physical health [18], [19].

Individuals cope with discriminatory experiences in a number of ways. A growing literature links weight bias with a number of coping behaviors, including problematic eating [6], [20] and avoidance of physical activity [7], [21]. For example, high school students who report feeling negative emotions due to weight-based victimization are more likely to cope by avoiding physical activity, including gym class, and are more likely to report increased food consumption [22]. This effect is not limited to high school students; adults who believe the negative stereotypes of obesity are true are more likely to refuse to diet and binge eat [20]. Thus, one coping mechanism for individuals who experience weight discrimination is to engage in the behaviors that are conducive to obesity.

As such, weight bias has been linked to factors that contribute to weight gain, but it has yet to be associated with actual changes in obesity over time. To that end, the present study tests whether reported weight discrimination is associated with becoming obese and remaining obese over a four-year period in a national sample of American adults. We hypothesize that among participants who are not obese at baseline, those who experience weight discrimination will be at greater risk for becoming obese by follow-up. Likewise, we hypothesize that among participants who are obese at baseline, those who experience weight discrimination will be at greater risk of remaining obese at follow-up. We also examine whether this effect generalizes to discrimination based on other characteristics (e.g., sex, race); we expect it to be specific to weight discrimination.

## Methods

### Study Design

Participants were drawn from the Health and Retirement Study (HRS), a nationally representative longitudinal study of Americans ages 50 and older [23]. HRS participants are re-interviewed every two years. Starting in 2004, participants in the enhanced face-to-face interview received a psychosocial questionnaire that they completed and returned by mail to the University of Michigan. Starting in 2006, this questionnaire included items about the experience of different types of discrimination, including weight (see below). We used the 2006 assessment as the baseline, since discrimination was first measured in this assessment. We used the obesity data from the 2010 assessment as the follow-up to have the longest longitudinal interval between assessments. A total of 6,157 participants (58.6% female) completed the discrimination measure and had weight and height at both the 2006 and 2010 assessments. These participants were, on average, 66.51 (*SD* = 10.04) years old, had an average of 12.83 (*SD* = 2.92) years of education, and were 84.7% white, 12.8% African-American, and 2.5% other ethnicities (self-reported). Human subjects approval for the HRS was obtained through the Institutional Review Board at the University of Michigan. HRS data is publically available for download at http://hrsonline.isr.umich.edu/.

### Obesity Status

Body Mass Index (BMI) was derived in the typical way (kg/m^2^) from reported weight and height. Participants were classified as obese (BMI≥30) or non-obese (BMI<30) at both the baseline and follow-up assessments. Of the 4,193 participants who were not obese at baseline, 357 (5.8% of the total sample) became obese by follow-up, and, of the 1,964 participants who were obese at baseline, 1,618 (26.3% of the total sample) remained obese at follow-up.

Most participants (*N* = 4,663) also had their weight and height measured by trained interviewers at both the 2006 and 2010 assessments. When BMI was derived from measured weight and height, 2,850 participants were not obese at baseline, and of those, 339 participants (5.3% of the total measured sample) became obese by follow-up. Of the 1813 participants who were obese at baseline, 1551 (24.3% of the total measured sample) remained obese at follow-up.

### Discrimination

Participants rated their experience of everyday discrimination [24] and then attributed those experiences to a number of personal characteristics, including weight [25]. In addition to weight, participants could also have attributed those experiences to their ancestry, sex, race, age, physical disability, other aspects of physical appearance, and/or sexual orientation. A total of 513 participants (8%) reported that they had been discriminated against because of their weight. Although single-item measures are not ideal, they have been used successfully to examine the effect of race [26] and sex [27] discrimination on smoking, to track trends in weight discrimination over time [2], and to document the correlates of weight bias [28].

### Statistical Analysis

We used logistic regression to test for the association between weight discrimination and change in obesity status. Weight discrimination was entered into a logistic regression to predict which participants, who were not initially obese at baseline, would become obese by follow-up and whether it would predict if obese participants at baseline would remain obese at follow-up. All analyses controlled for age, age squared, sex, ethnicity, and education. We likewise used logistic regression to examine whether discrimination based on other characteristics (race, ancestry, sex, age, physical disability, other aspects of physical appearance, sexual orientation) was associated with becoming obese or remaining obese over the follow-up period, controlling for the same demographic characteristics. We also reran all analyses controlling for baseline BMI.

We did a number of follow-up analyses to examine the robustness of the initial findings. First, we repeated the logistic regressions using BMI derived from measured weight and height. Second, for the becoming-obese analyses, we reran the analyses limiting the sample to those who were in the normal weight range at baseline and again limiting it to those who were in the overweight range (BMI ≥25 and <30) at baseline. Third, we tested for interactions between weight discrimination and age, sex, ethnicity, and education to test whether these demographic factors moderated the association between weight discrimination and obesity status. We used SPSS 21 for all analyses.

## Results

Weight discrimination was associated with becoming obese between baseline and follow-up: Among participants who were not obese at baseline, those who reported weight discrimination were approximately 2.5 times more likely to be obese by follow-up than those who did not report weight discrimination (see Table 1). This effect was specific to weight discrimination; the other types of discrimination were largely unrelated to reported obesity. That is, none of the other types of discrimination assessed were associated with becoming obese between the two assessments. The one exception was age discrimination, which was associated with becoming obese, but this association was reduced to non-significant once baseline reported BMI was included in the analysis. In contrast, although attenuated, the association between weight discrimination and becoming obese remained significant even after accounting for baseline reported weight (Odds Ratio [OR] = 1.72, 95% Confidence Interval [CI] = 1.01–2.95). The findings were similar when we used BMI derived from measured weight and height.

**Table 1:** Odds Ratios (95% Confidence Interval) for Discrimination on Obesity over Four Years.

Discrimination	%	Became Obese	Remained Obese
Weight	8.0	2.54 (1.58–4.08)** a	3.20 (2.06–4.97)** a
Race	10.1	.83 (.53–1.30)	1.18 (.78–1.80)
Ancestry	6.2	1.25 (.81–1.95)	.73 (.46–1.16)
Sex	13.6	1.20 (.88–1.64)	1.30 (.89–1.90)
Age	30.0	1.31 (1.03–1.66)*	.89 (.69–1.15)
Physical Disability	5.9	.95 (.58–1.56)	1.06 (.68–1.67)
Appearance	5.9	.91 (.55–1.48)	.69 (.45–1.07)
Sexual Orientation	1.7	1.92 (.95–3.89)	1.65 (.58–4.73)

*Note*. *N* = 6,157. All analyses controlled for age, age squared, sex, ethnicity, and education.

* *p*<.05.

** *p*<.01.

a Significant after controlling for baseline body mass index.

We next examined whether the effect of discrimination on risk of obesity was similar for normal weight versus overweight participants. The risk of obesity was similar when the sample was limited to participants who reported being overweight at baseline (OR = 2.10, 95% CI = 1.24–3.56) but was much greater for participants who reported normal weight at baseline (OR = 6.13, 95% CI = 1.62–23.24). Interestingly, a slightly different pattern emerged when the analyses were based on measured BMI. When the sample with measured weight and height was limited to participants who were overweight at baseline, the risk of obesity was a little stronger but essentially the same (OR = 2.18, 95% CI = 1.04–4.45). In contrast, when this sample was limited to normal weight participants at baseline, there was not enough data for the analysis: of the 14 participants in the normal weight category who reported weight discrimination, none became obese.

Similar to weight gain, weight discrimination was associated with remaining obese over the period between the two assessments (see Table 1). That is, those who experienced discrimination based on their weight were over three times more likely to remain obese at follow-up, rather than drop below the obesity threshold, than those who did not experience such discrimination. Also similar to becoming obese, this association was specific to weight discrimination; none of the other types of discrimination were associated with remaining obese, and this association remained significant after controlling for baseline reported BMI (OR = 1.69, 95% CI = 1.06–2.70). The effect was similar when we used BMI derived from measured weight and height.

Finally, none of these associations were moderated by any of the demographic factors, which indicated that the association between weight discrimination and risk of becoming and remaining obese did not vary by age, sex, ethnicity, or education.

## Discussion

The mental health correlates of weight discrimination have been well documented; the present research indicates that there are also significant physical health correlates of such discrimination. These findings follow from the related literature on racial discrimination that shows that discrimination increases risk of hypertension [17], severe coronary obstruction [29], and elevated inflammatory markers, such as C-reactive protein [30]. Like other forms of discrimination, body weight is a highly visible, personal characteristic that can evoke strong stereotypes and strong reactions from others.

There are both behavioral and physiological mechanisms that may contribute to the relation between discrimination and obesity. Weight discrimination is associated with behaviors that increase risk of weight gain, including excessive food intake and physical inactivity. There is robust evidence that internalizing weight-based stereotypes [20], teasing [31], and stigmatizing experiences [32] are associated with more frequent binge eating. Overeating is a common emotion-regulation strategy, and those who feel the stress of stigmatization report that they cope with it by eating more [22]. Individuals who endure stigmatizing experiences also perceive themselves as less competent to engage in physical activities [33] and are thus less willing to exercise and tend to avoid it [21]. Finally, heightened attention to body weight is associated with increased negative emotions and decreased cognitive control [34]. Increased motivation to regulate negative emotions coupled with decreased ability to regulate behavior may further contribute to unhealthy eating and behavioral patterns among those who are discriminated against.

Physiological mechanisms may also contribute to the relation between discrimination and unhealthy eating and weight gain. Psychological stress, particularly stress that involves heightened public awareness, engages the hypothalamic-pituitary-adrenal (HPA) axis and triggers cortisol release [35], [36], and experimental administration of glucocorticoids has been found to increase food intake [37]. Further, individuals with high cortisol reactivity tend to choose calorie-dense, high-fat foods following a social stress test [38]. In addition to the secretion of cortisol, activation of the HPA axis also stimulates the release of endogenous opioids, which serve to regulate HPA activity [39]. The release of such opioids stimulates intake of palatable foods, and palatable foods likewise encourage opioid release [40]; a cycle may thus ensue that reinforces intake of high-fat, calorie-dense foods as a way to cope with stress.

The basic results were similar across analyses using either self-reported or measured weight and height. An interesting divergence emerged for the follow-up analyses on risk of becoming obese for participants who were normal weight at baseline. For participants who fell in the normal weight range, there was a strong effect of discrimination on risk of self-reported obesity. When obesity was derived from measured weight and height, however, none of the normal weight participants who reported discrimination became obese by follow-up. As with other factors, reliance on self-reported weight and height may exaggerate the effect of interest [41]. Of note, in contrast to normal weight participants, the effect of weight discrimination on risk of obesity for participants who were overweight at baseline was quite similar across reported and measured weight and height. Thus, weight discrimination has the greatest consequences for risk of obesity among those who are overweight or obese, which are the groups most likely to experience weight discrimination.

This study had several strengths, including a large and fairly diverse sample and a 4-year longitudinal interval. Future research could address some limitations. First, weight discrimination was assessed with a single-item measure. Although previous research has successfully used single-item measures to examine health-related correlates of discrimination [26] and to document the correlates of weight bias [28], a more detailed scale would provide more information on the relation between weight discrimination and the development of obesity. In addition, the sample was drawn from an older population, which may have different experiences with weight discrimination than younger populations. It would be interesting to replicate these effects in young adults. Finally, we did not test potential mechanisms that may lead from discrimination to obesity. Future research could test factors such as emotional eating and cortisol as mechanisms of this association.

Given the complex etiology of obesity, creative approaches that span diverse disciplines are needed to combat its spread. Weight discrimination, which is often justified because it is thought to help encourage obese individuals to lose weight [1], can actually have the opposite effect: it is associated with the development and maintenance of obesity. Such discrimination is one social determinant of health that may contribute to inequities in employment, relationships, healthcare delivery, and body weight.
